# The Development of a Standard Reference Material for Calibration of the University of Pittsburgh Smoke Toxicity Method for Assessing the Acute Inhalation Toxicity of Combustion Products

**DOI:** 10.6028/jres.097.006

**Published:** 1992

**Authors:** Barbara C. Levin, Yves Alarie, Maryanne F. Stock, Susannah B. Schiller

**Affiliations:** National Institute of Standards and Technology, Gaithersburg, MD 20899; University of Pittsburgh, Pittsburgh, PA 15261; National Institute of Standards and Technology, Gaithersburg, MD 20899

**Keywords:** combustion, combustion products, inhalation, nylon, nylon 6/6, SRM, standard reference material, toxicity tests, University of Pittsburgh

## Abstract

A standard reference material (SRM 1049) has been developed for the University of Pittsburgh smoke toxicity method. SRM 1049 is a nylon 6/6 and has the molecular structure of [–NH(CH_2_)_6_NHCO(CH_2_)_4_CO–]*_n_*. This SRM is for calibrating the apparatus and providing confidence that the method is being conducted in a correct manner and that the equipment is functioning properly. The certified figure of merit is a *LC*_50_ value plus its 95% prediction interval which were calculated and found to be 4.4 + 3.4 g. The 95% prediction interval indicates the range in which the next determined *LC*_50_ value would be expected to fall. Thus, if an investigator were to test this SRM under their laboratory conditions according to the specifications of the University of Pittsburgh test procedure and found the *LC*_50_ value fell within the certified 95% prediction interval, the probability is good that the test is being conducted correctly.

## 1. Introduction

In 1973, The National Commission on Fire Prevention and Control issued the report “America Burning” [1] which noted that most fire victims die from inhaling smoke and toxic gases. This information served as one of the motivating forces in the development and testing of many smoke toxicity test procedures [2]. In 1983, 13 of these published methods were evaluated by Arthur D. Little, Inc. to assess the feasibility of incorporating combustion toxicity requirements for building materials and finishes into the building codes of New York State [3]. On the basis of seven different criteria, only two methods were found acceptable. These two methods were the flow-through smoke toxicity method developed at the University of Pittsburgh [4,5] and the closed-system cup furnace smoke toxicity method [6] developed at the National Institute of Standards and Technology (NIST).

Based on the results of the A. D. Little report, the state of New York under Article 15, Part 1120 of the New York State Fire Prevention and Building Code decided that building materials and finishes should be examined by the method developed at the University of Pittsburgh and that the results be filed with the state [7]. It is important to note, however, that although the results are filed, the state of New York does not regulate any materials or products based on the results of toxicity testing. It is also important to note that, at the present time, no smoke toxicity method has been accepted as a standard test by ASTM or any other national or international scientific or technical society designed to develop standard test procedures. Thus, the development of other smoke toxicity methods is still being actively pursued.

Three methods currently under development are the University of Pittsburgh II radiant furnace method [8,9,10], a radiant furnace smoke toxicity protocol [11,12] which is being developed at NIST and the National Institute of Building Sciences (NIBS) toxic hazard test method [13,14]. Although these methods differ significantly in numerous characteristics, all three use radiant heat to decompose materials. Documentation of the relevance and accuracy of the radiant methodology may be found in Refs. [11] and [12].

Over the past decade, the number of smoke toxicity test apparatus users has increased. A number of Federal agencies, industrial laboratories, and testing companies are capable of conducting both the University of Pittsburgh and the cup furnace smoke toxicity test procedures. Although there are no state or federal regulations, the results of these smoke toxic potency tests, along with the results of other material flammability tests, are being used in the decision making process regarding material selection and overall fire hazard. Therefore, it is necessary to assure that such testing devices are installed and employed properly both by those laboratories currently conducting these tests and by new laboratories that enter the field. To help assure the reproducibility of results between laboratories, NIST has developed two standard reference materials (SRMs), one which can be used to calibrate the University of Pittsburgh smoke toxicity method (SRM 1049) and another SRM (SRM 1048) which can be used to calibrate the cup furnace smoke toxicity method [15]. *It is important to note that these SRMs were not selected to represent the toxic potency of the combustion products of an “average” material and are not designed to be used for the comparison of the relative toxic potency of the combustion products of test materials. Therefore, toxic potency of the smoke from a test material should not be compared to the toxic potency of the smoke from these SRMs.*

The following criteria were used in the selection process of the University of Pittsburgh smoke toxicity SRM:

1. The material should have reproducible burning characteristics (i.e., the material must be homogeneous),
2. The material should produce combustion products whose toxic potency values are within the range where the values for some other materials are found.
3. Upon combustion, toxic gases in addition to CO should be generated and contribute to the lethal atmospheres, and
4. The selected material should generate combustion products which cause deaths during the animal exposures. The University of Pittsburgh method does not specify the post-exposure observation of the test animals other than an immediate 10 min period following the exposure.

The polymer nylon 6/6, whose characteristics fit the above criteria, was selected for the University of Pittsburgh smoke toxicity SRM. An intralaboratory evaluation (performed at the University of Pittsburgh, Pittsburgh, PA) and an interlaboratory evaluation (carried out by Anderson Laboratories, Inc., Dedham, MA, Southwest Research Institute, San Antonio, TX, University of Pittsburgh, U.S. Testing Co., Inc., Hoboken, NJ, and Weyerhaeuser Co., Longview, WA) were conducted to determine the repeatability of results within a laboratory and reproducibility of results between laboratories, respectively. When the intra- and interlaboratory evaluations showed good repeatability and reproducibility of results with nylon 6/6, additional material of a single lot number was ordered for certification as an SRM. Further testing of the new lot was conducted by University of Pittsburgh and Anderson Laboratories to provide the data necessary for the development of the final certified SRM.

This paper documents the research and development of SRM 1049 which will be used to calibrate the University of Pittsburgh smoke toxicity test procedure and will help assure that the apparatus is performing correctly. To use SRM 1049 in the calibration of the test procedure, a laboratory would determine the *LC*_50_ value of the SRM according to the published University of Pittsburgh test procedure [4,5,7] and compare it with the certified *LC*_50_ value and its 95% prediction interval.^1^ If the experimental value obtained by the laboratory falls within the 95% prediction interval of the certified *LC*_50_ value of this SRM, the investigator can be confident that the method is being conducted correctly.

## 2. Materials and Methods

### 2.1 Materials

Two separate lots of nylon 6/6 [poly(hexamethylene adipamide)] with a molecular structure of [–NH(CH_2_)_6_NHCO(CH_2_)_4_CO–]*_n_* were obtained from Aldrich Chemical Co.^2^ The first sample of nylon 6/6 (lot 80078; hereafter referred to as lot 1) was tested by the University of Pittsburgh and four other laboratories. Based on the results of this interlaboratoiy evaluation of the University of Pittsburgh smoke toxicity method, nylon 6/6 was found to be a suitable candidate for an SRM. A second batch of nylon 6/6 was ordered for certification purposes. The second batch of nylon 6/6 was in the same pellet form and had the same manufacturer specifications as the first batch, but had a different lot number (lot 08015; hereafter referred to as lot 2). Each bottle containing 1 kg of nylon 6/6 (lot 2) was randomly numbered when received at NIST. Samples of four bottles, Nos. 10, 20, 30, and 40, from lot 2 were used for certification purposes.

### 2.2 Animals

Swiss Webster mice weighing between 22 and 28 g were used for this study. They were allowed to acclimate to the laboratory conditions for approximately 1 week. Animals were group housed with free availability of food and water. Only animals appearing healthy were used for the study.

### 2.3 Experimental Method

**Fig. 1:**
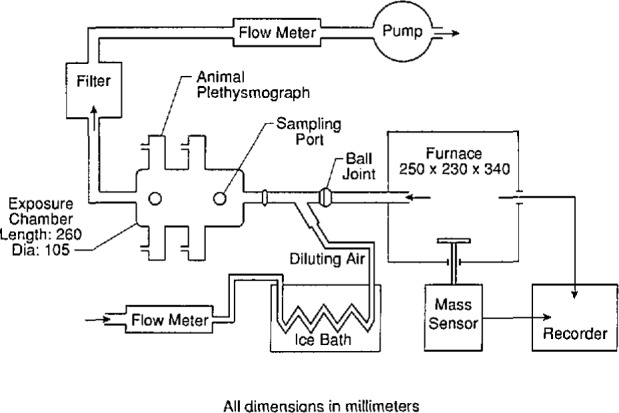
Schematic of the experimental test system used to decompose the sample and expose the animals.

The University of Pittsburgh smoke toxicity method to evaluate the acute inhalation toxicity of combustion products was developed by Alarie and Anderson [4,5]. The experimental arrangement is illustrated in Fig. 1. In this method, the sample is placed on a load sensor in the furnace at room temperature. The temperature is then increased at the rate of 20 °C/min. The temperature at which the sample begins to decompose, the rate at which the material decomposes as the temperature increases, and the time of ignition (i.e., flaming) of the sample are recorded.

Animal (head only) exposure to the thermal decomposition products is started when 1.0% mass loss of the material occurs. Four Swiss Webster male mice between 22 and 28 g in weight are exposed in each experiment. The exposure is 30 min in duration. The toxicological endpoint is death which occurs during the 30 min exposure period and a 10 min post-exposure observation period. The amount of material which releases enough smoke to cause either 0, 25, 50, 75, or 100% of the animals to die is used to calculate the *LC*_50_ values. During the animal exposures, the major combustion products, carbon monoxide (CO), carbon dioxide (CO_2_), and reduced oxygen (O_2_) are continuously monitored.

In the evaluation and development of SRM 1049 (i.e., data presented in this paper), the *LC*_50_ values and their 95% confidence limits were determined by the statistical method of Weil [16]. At the University of Pittsburgh, CO, CO_2_, and O_2_ were measured continuously by a Miran 1A infrared gas analyzer^3^, a Beckman LB-2 medical gas analyzer, and a Beckman OM-11 oxygen analyzer, respectively. At Anderson Laboratories, CO and CO_2_ were measured continuously by Horiba nondispersive infrared gas analyzers and O_2_ was measured continuously by a Lynn electrochemical oxygen analyzer. Even though the material contained nitrogen, concentrations of hydrogen cyanide generated during these experiments were not measured.

### 2.4 Comparison Factors in the Development of this SRM

#### 2.4.1 Interlaboratory Evaluation

To ascertain the reproducibility across different laboratories, four laboratories (in addition to the University of Pittsburgh) were asked to participate in an interlaboratory evaluation of nylon 6/6 (lot 1). These laboratories were Anderson Laboratories (Dedham, MA), Southwest Research Institute (San Antonio, TX), U.S. Testing Co., Inc. (Hoboken, NJ), and Weyerhaeuser Company (Longview, WA).

#### 2.4.2 Intralaboratory Evaluation

The University of Pittsburgh examined the repeatability of the *LC*_50_ for both lots of nylon 6/6. Three separate *LC*_50_ values were determined for nylon 6/6 (lot 1) and four *LC*_50_ values for lot 2. In addition, two separate samples from lot 2 were sent to Anderson Laboratories which determined two *LC*_50_ values.

### 2.5 Statistical Analysis of Results

For this SRM, two types of statistical uncertainties, a 95% *confidence* interval and a 95% *prediction* interval, were determined. The 95% confidence interval defines the precision with which the true endpoint (the *LC*_50_) is known; whereas, the 95% prediction interval provides the numerical bound in which the next *LC*_50_ should fall if the experiments are conducted correctly. Unlike a 95% confidence interval, a 95% prediction interval does not get appreciably narrower if more laboratories participate in the study, since the 95% prediction interval will always be larger than the interlaboratory standard deviation. The difference between these two intervals is illustrated below for the simplified case in which each of *“n”* laboratories would determine one *LC*_50_ value.

The uncertainty based on a 95% confidence interval for the true *LC*_50_ is shown in Eq. (1)

$$t_{n-1}(0.025)\sqrt{\frac{\text{interlab variance}}{n}}$$ (1)

where *t_n_*_−1_(0.025) is the appropriate cutoff from the student’s *t* distribution for a two sided interval with a 95% confidence level [17].

The uncertainty based on a 95% prediction interval, using data from the same simple case as above, would be determined by Eq. (2) [18]. This calculation incorporates the variance of a single new determination of the *LC*_50_ value plus the variance of the mean.

$$t_{n-1}(0.025)\sqrt{\frac{\left(n+1\right)}{n}\;\text{interlab variance}}$$ (2)

Since we had limited data on the SRM material (only two laboratories evaluated it), we estimated the variability in the certified *LC*_50_ using data from the interlaboratory study (on lot 1) as well as the data on the SRM (lot 2). Analysis of these data showed that the variability between laboratories was larger than the variability within the laboratories. These data could be pooled since the two lots of nylon 6/6 were considered fairly similar based on the material composition and the expectation that the measurement errors made by the laboratories would follow the same distribution for both lots. In addition, an Analysis of Variance indicated that there was no statistically significant difference between the mean *LC*_50_ of lot 1 and that of lot 2. The interlaboratory and intralaboratory variance components were estimated from an analysis of variance on both materials simultaneously. The variance of the mean *LC*_50_ value for the SRM is given in Eq. (3):

$$\frac{1}{2}\text{interlab variance}+\frac{3}{16}\;\text{within-lab variance}$$ (3)

and the effective degrees of freedom for this variance are 4.9. Therefore, the uncertainty based on a 95% confidence interval for the mean is shown in Eq. (4):

$$t_{4.9}(0.025)\sqrt{\frac{1}{2}\text{interlab variance}+\frac{3}{16}\text{within-lab variance}}$$ (4)

The variance of a single new *LC*_50_ plus the variance of the mean is shown in Eq. (5):

$$\frac{3}{2}\text{interlab variance}+\frac{19}{16}\text{within-lab variance}$$ (5)

and the effective degrees of freedom for this sum are 5.3. Therefore, the uncertainty based on a 95% prediction interval is seen in Eq. (6):

$$t_{5.3}(0.025)\sqrt{\frac{3}{2}\text{interlab variance}+\frac{19}{16}\text{within-lab variance}}$$ (6)

## 3. Results

### 3.1 Interlaboratory Evaluation

The toxicological data for nylon 6/6 (lot 1) provided by the participating laboratories in the interlaboratory evaluation are given in Table 1.

### 3.2 Intralaboratoty Evaluation

**Fig. 2:**
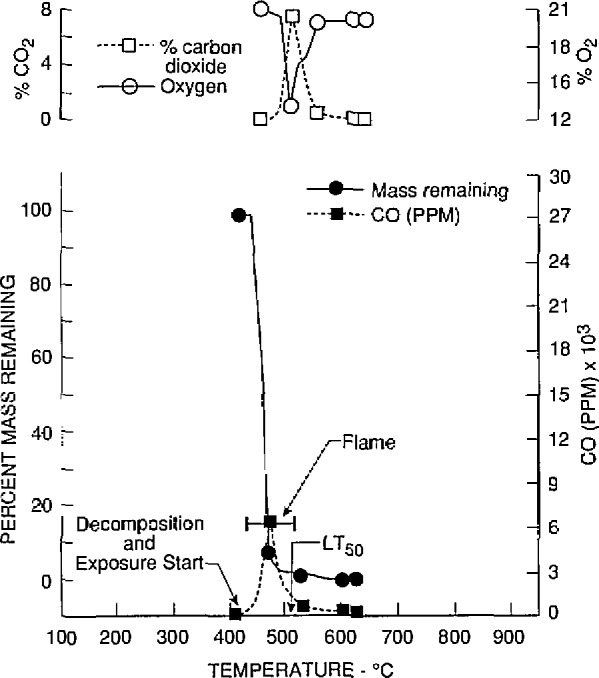
Gas concentrations and percent mass remaining as furnace temperatures increased during the decomposition of nylon 6/6 (lot 2) from bottle No. 10. The initial mass of nylon 6/6 was 3.6 g (the *LC*_50_ value).

**Fig. 3:**
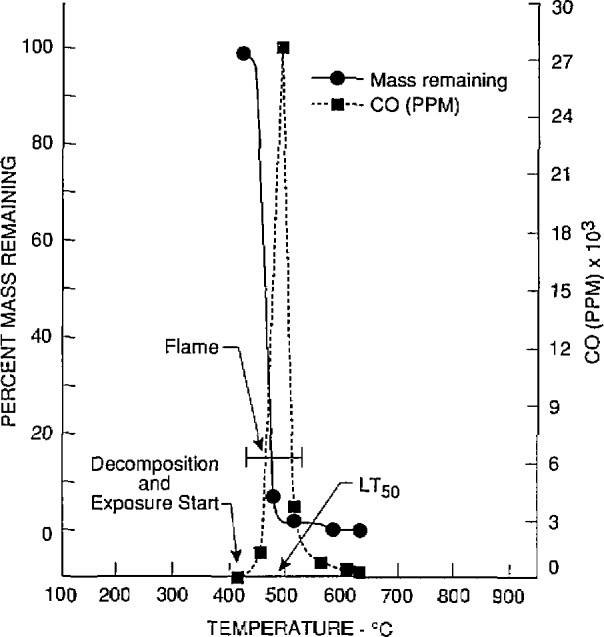
Carbon monoxide cancentrations and percent mass remaining as furnace temperatures increased during the decomposition of nylon 6/6 (lot 2) from bottle No. 20. The initial mass of nylon 6/6 was 3.9 g (the *LC*_50_ value).

**Fig. 4:**
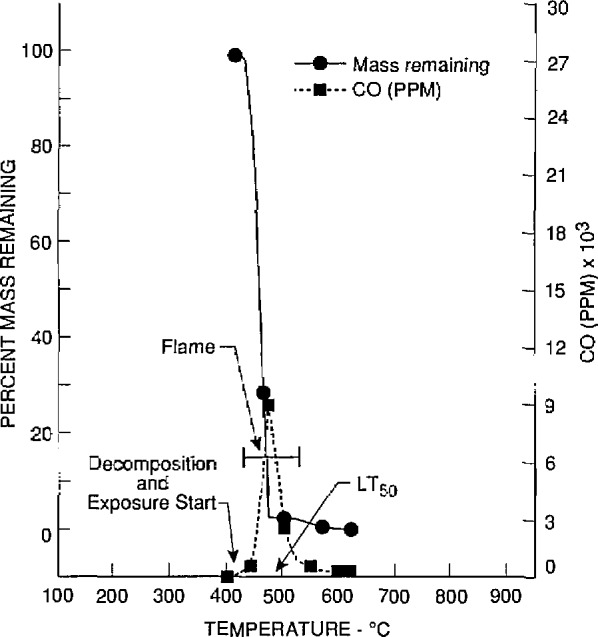
Carbon monoxide concentrations and percent mass remaining as furnace temperatures increased during the decomposition of nylon 6/6 (lot 2) from bottle No. 30. The initial mass of nylon 6/6 was 3.6 g (the *LC*_50_ value).

**Fig. 5:**
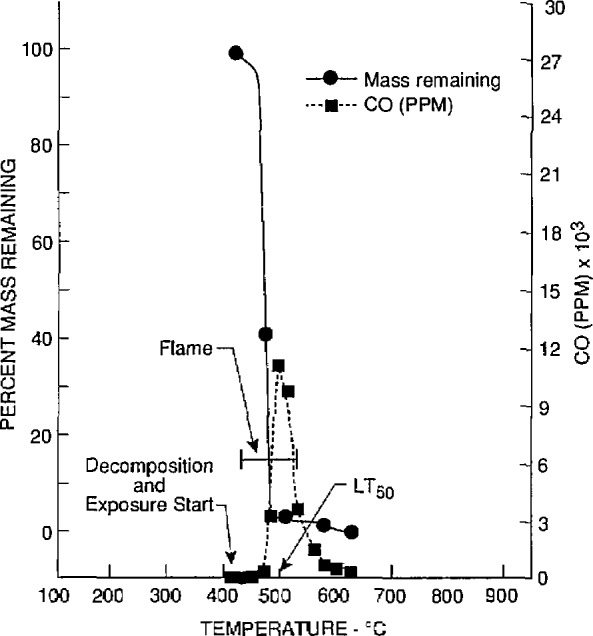
Carbon monoxide concentrations and percent mass remaining as furnace temperatures increased during the decomposition of nylon 6/6 (lot 2) from bottle No. 40. The initial mass of nylon 6/6 was 3.9 g (the *LC*_50_ value).

The toxicological and chemical data for nylon 6/6 (lot 2) obtained at the University of Pittsburgh and Anderson Laboratories are given in Tables 2 and 3, respectively. The *LC*_50_ values of four separate samples (bottles 10, 20, 30, and 40) of lot 2 were determined by the University of Pittsburgh. Two additional *LC*_50_ values were obtained for two samples of lot 2 (bottles 20 and 30) by Anderson Laboratories. The material mass loss, evolution of CO and CO_2_, and reduction of O_2_ found in tests conducted by the University of Pittsburgh at or close to the *LC*_50_ values are illustrated in Figs. 2 to 5. These four figures also show the temperatures at which the material decomposition began and when flaming ignition occurred, and the temperatures at which animal exposure was initiated as well as the temperature at the time 50% of the animals died (*LT*_50_).

## 4. Discussion

The development of a standard reference material requires the statistical determination of a certified value (in this case, the *LC*_50_ of the material) and an uncertainty which informs the user how well the certified value is known. For most standard reference materials, this uncertainty is defined by the 95% *confidence* interval, which, in the example of SRM 1049, would tell the user how precisely the true *LC*_50_ value is known (with 95% confidence). However, for this SRM, we have determined the 95% *prediction* interval rather than the 95% confidence interval. The 95% prediction interval tells the user the numerical bound in which the next *LC*_50_ value should fall assuming the experiments are conducted correctly and instruments are functioning properly. A confidence interval is narrower than a prediction interval and will get narrower as the number of participating laboratories increases. Even if the user’s system is operating correctly and his precision is comparable to the precision found in the research reported here, the next *LC*_50_ value could reasonably fall in a much larger range than that given by the confidence interval. On the other hand, the 95% prediction interval will not get smaller than the precision of a single measurement and thus allows the user to judge if the next experimental value obtained in his laboratory is in the right range.

The mean *LC*_50_ value of SRM 1049 (nylon 6/6; lot 2) is 4.4 g; its 95% confidence interval is ±1.9 g; whereas, its 95% prediction interval is ± 3.4 g. This 95% prediction interval incorporates the variability from both the within-laboratory experiments and the interlaboratory evaluation of nylon 6/6. Therefore, if the user’s precision is comparable to the precision found in the data in this report and the test procedure is being conducted correctly, the 95% prediction interval should include the user’s next *LC*_50_ measurement.

## 5. Conclusions

A standard reference material (SRM 1049) has been developed for calibration of the University of Pittsburgh smoke toxicity method for assessing the acute inhalation toxicity of combustion products. The certified material is a nylon 6/6 and the certified *LC*_50_ value was based on four series of tests conducted at the University of Pittsburgh and two series of tests conducted at Anderson Laboratories. The 95% prediction interval is based on the variability of results found in both interlaboratory and intralaboratory evaluations. The certified *LC*_50_ value and 95% prediction interval is 4.4 ±3.4 g. If a laboratory were to test this SRM under their conditions in their apparatus and found the *LC*_50_ value to fall within the certified 95% prediction interval, the probability is good that their equipment is functioning appropriately and that the test is being conducted correctly.

## Figures and Tables

**Table 1:** Interlaboratory evaluation of nylon 6/6 (lot 1) toxicological data

Laboratory	*LC*_50_ values (g)	Mean *LC*_50_ values (g)
Univ. of Pittsburgh	4.8 (4.2–5.3) a	5.2
	5.2 (4.8–5.7)	
	5.7 (5.3–6.2)	
1	3.6 (3.6–3.6)	3.8
	3.7 (3.1–4.6)	
	4.1 (3.3–5.0)	
2	6.1 (6.0–6.2)	6.6
	7.1 (6.7–8.0)	
3	5.4 (4.8–6.1)	
	5.4 (4.8–6.1)	
4	4.3 (3.6–5.4)	4,3
Overall mean ±95% confidence interval^b^		5.1 ±1.2

a 95% confidence limits of the *LC*_50_ values.

b Mean of all the laboratories’ mean values. The 95% confidence interval incorporates both the within-laboratory and the between-laboratory variation.

**Table 2:** Intralaboratory evaluation of nylon 6/6 (lot 2) toxicological data

Laboratory	Bottle No.	*LC*_50_ values (95% confidence limits) (g)	Mean *LC*_50_ value (g)
U. Pitt.	10	3.6 (ND)	
	20	3.9 (3.7–3.9)	3.7
	30	3.6 (3.5–3.7)	
	40	3.9 (3.9–4.0)	
Anderson	20	5.1 (4.5–5.8)	5.1
	30	5.15^a^ (5.1^b^–5.2^c^)	
Overall mean ±95% confidence interval			4.4 ±1.9^d^

a Midpoint between values in parenthesis

b No deaths at this mass loading

c 100% deaths at this mass loading

d The overall mean is based on the mean values from the two laboratories; The 95% confidence interval incorporates both the within and between laboratory variation.

ND–not determined.

**Table 3:** Intralaboratoty evaluation of nylon 6/6 (lot 2) physical and chemical data^a^

Laboratory	Bottle No.	Temp. initial exposure(°C)	Temp. during flaming (°C)	Time of flaming” (min)	Max CO (%)	Time max CO (min)	Max CO_2_ (%)	Time max CO_2_ (min)	MinimumO_2_ (%)	Time minimum O_2_ (min)
Univ. of Pittsburgh	10	410	431–513	1.5	0.64	3	7.5	3	13	3
	20	415	448–531	1.7	2.8	3.5	ND	ND	ND	ND
	30	410	447–536	2.0	0.90	3.5	ND	ND	ND	ND
	40	415	441–531	1.3	1.1	4.5	ND	ND	ND	ND
Anderson	20	409	434–612	NR	1.2	NR	7.0	NR	11.6	NR
	30	401	435–601	NR	1.2	NR	7.6	NR	11.8	NR

a Values in table are from experiments conducted at a concentration equivalent to the *LC*_50_ value.

b Time that material started flaming where the beginning of the animal exposure is 0 time.

ND–not determined.

NR–not reported.

## Footnotes

1 In the University of Pittsburgh smoke toxicity method, the *LC*_50_ is defined as the statistical estimate of the amount of material (in grams) which, when placed into the furnace, would cause 50% of the exposed mice to die within the 30 min exposure and the 10 min post-exposure observation period.

2 Certain commercial equipment, instruments, or materials are identified in this paper to specify adequately the experimental procedure. Such identification does not imply recommendation or endorsement by the National Institute of Standards and Technology, nor does it imply that the materials or equipment identified are necessarily the best available for the purpose.

3 The absolute gas concentrations reported in this paper depend upon the analytical instruments used in measuring the specific gas species and the instruments’ response times.
